# A Prognostic Model for Senescence-Related LncRNA in a Novel Colon Adenocarcinoma Based on WGCNA and LASSO Regression

**DOI:** 10.3390/biomedicines13051088

**Published:** 2025-04-30

**Authors:** Yichu Huang, Guangtao Min, Hongpeng Wang, Lei Jiang

**Affiliations:** 1 The First School of Clinical Medicine, Lanzhou University, Lanzhou 730000, China; 2 Department of General Surgery, The First Hospital of Lanzhou University, Lanzhou 730000, China; 13919256670@163.com (G.M.);

**Keywords:** colon adenocarcinoma, LASSO, WGCNA, senescence-related lncRNA

## Abstract

**Objective**: This study aims to develop a prognostic model based on senescence-related long non-coding RNAs (lncRNAs) to predict the prognosis of patients with colon cancer and enhance their survival rates. **Method**: Differential expression analysis and Pearson correlation were employed to identify senescence-related lncRNAs in colon cancer. A risk prognosis model was constructed using univariate Cox regression analysis and Least Absolute Shrinkage and Selection Operator (LASSO) regression analysis. The reliability of this model was validated through survival analysis, receiver operating characteristic (ROC) curves, bar charts, and calibration curves. Additionally, the relationship between the prognostic model, immune microenvironment, and drug sensitivity was explored. **Results**: A risk prognosis model comprising eight senescence-related lncRNAs (LINC02257, AL138921.1, ATP2B1-AS1, AC005332.7, AC007728.3, AC018755.4, AL390719.3, and THCAT158) was successfully established, demonstrating strong performance in predicting the overall survival rates of colon cancer patients (AUC = 0.733). A significant correlation was observed between the senescence-related lncRNA prognostic model and the tumor microenvironment, immune cell infiltration, and drug sensitivity (*p* < 0.05). **Conclusions**: The senescence-related lncRNA prognostic model developed in this work can accurately forecast the prognosis of colon cancer patients, offering new insights for personalized treatment approaches in colon cancer.

## 1. Introduction

Colon cancer is a type of malignant tumor influenced by various genetic and environmental factors that pose a significant threat to human health. In recent years, factors such as economic development, changes in dietary habits and lifestyles, and the rapid aging of the population have contributed to a yearly rise in both the incidence and mortality rates associated with colon cancer. Global cancer data from 2020 show that colon cancer has the third highest incidence rate and the highest case fatality rate among malignant tumors [1,2]. The symptoms of this disease often develop gradually, and this, together with the rate of endoscopic screenings being relatively low in China, means most patients have lost the best surgical opportunity when seeking treatment [3,4]. Consequently, identifying novel diagnostic and therapeutic targets for colon cancer is crucial for enhancing its diagnosis and treatment, as well as improving patient outcomes.

Aging represents the cellular response to various stress signals and serves to protect cells from unnecessary harm. In the context of cancer, aging has a dual function: it acts as a tumor suppressor by inhibiting the proliferation of damaged cells, while simultaneously promoting cancer by fostering an inflammatory environment. Furthermore, cancer cells can also undergo senescence responses. This presents both challenges and opportunities in cancer treatment through a sequential “one-two punch” strategy employing pro-senescence therapy followed by senolytic therapy [5]. Long non-coding RNA (lncRNA) is a type of non-coding RNA that exceeds 200 nucleotides in length. It functions biologically by regulating gene expression and is crucial in the development and progression of cancer [6]. LncRNAs play pivotal roles in regulating multiple pathological processes in colon cancer, including cell proliferation, apoptosis, cell cycle progression, migration, epithelial–mesenchymal transition (EMT), cancer stem cell maintenance, and resistance to therapeutic interventions [7]. The E2F1-reactive lncRNA LIMp27 competes with p27 mRNA for binding to cytoplasmic hnRNP0, selectively downregulating p27 expression. This interaction results in passage through the G0/G1 phase of the cell cycle and promotes the proliferation, tumorigenicity, and therapeutic resistance of colon adenocarcinoma cells lacking p53 [8]. Investigating senescence-related lncRNAs in colon adenocarcinoma can enhance our understanding of the molecular mechanisms involved in the onset and progression of this cancer, while also paving the way for the development of novel intervention strategies.

## 2. Materials and Methods

### 2.1. Public Database Acquisition and Processing

We obtained mRNA sequencing and non-coding RNA sequencing data for TCGA-COAD patients from The Cancer Genome Atlas (TCGA) database (https://portal.gdc.cancer.gov/, accessed on 23 October 2024), along with clinical and pathological characteristics of 444 colon adenocarcinoma patients and 11 matched normal controls. Additionally, 90 senescence-related genes associated with colon adenocarcinoma were sourced from the published literature [9].

### 2.2. Screening of Senescence-Related lncRNAs

We conducted Pearson correlation analysis using the R packages “limma”, “ggplot2”, “ggalluvial”, and “dplyr” to evaluate the co-expression relationships between senescence-related genes and lncRNAs. The screening criteria were set as |correlation coefficient| (corFilter) > 0.4 and *p*-value < 0.05. A Sankey diagram was generated via the “ggalluvial” package to visualize the associations between senescence-related lncRNAs and their correlated genes.

### 2.3. Construction of a Prognostic Model for Senescence-Related lncRNA

Utilizing the R package “caret” (version 7.0-1), the adenocarcinoma cell dataset obtained from TCGA was randomly divided into training (n = 182) and testing (n = 273) sets. The reliability of the prediction model was validated using the testing set and the entire TCGA dataset. To further investigate senescence-related lncRNAs, a single-factor Cox regression analysis was performed, and the findings were illustrated in a forest plot. LASSO regression analysis was then employed to select the optimal group of lncRNAs for the development of a risk model. Subsequently, multivariate Cox regression analysis was conducted to create a prognostic model based on the selected lncRNAs. The risk score for each cancer patient was calculated using the equation: risk score = (∑βlncRNA × Exp_lncRNA). A chi-square test was used to assess whether there were statistically significant differences in clinical data between the training and testing sets. Additionally, the “ggplot2” package was used to generate a correlation heatmap depicting the relationship between senescence-related lncRNAs and senescence-related genes.

### 2.4. Validation of the Efficacy of Prognostic Models

Patients were categorized into high-risk and low-risk groups based on the median risk score. The “survival” package was utilized to assess whether there were significant differences in overall survival (OS) between these two groups through Kaplan–Meier survival curves. To validate the independence of the model, independent prognostic analyses were conducted considering factors such as patient age, tumor grade, and stage. Utilizing the patients’ survival status along with the “surminer” and “timeROC” packages, receiver operating characteristic (ROC) curves were generated and the area under the curve (AUC) was calculated. Additionally, a concordance index (C-index) curve was drawn to evaluate the model’s accuracy. Patients were further divided into early (stage I–II) and late (stage III–IV) stages based on tumor staging, and Kaplan–Meier survival curves were employed to compare the OS of high-risk and low-risk groups in both early- and late-stage patients.

### 2.5. Column Chart and Principal Component Analysis

Nomograms predicting 1-, 3-, and 5-year OS rates for colon cancer patients were constructed using the “rms” and “survival” R packages. The risk scores were incorporated into a comprehensive analysis with clinicopathological factors. Calibration curves based on the Hosmer–Lemeshow test were generated to evaluate the predictive accuracy of the nomograms. Furthermore, principal component analysis (PCA) was performed via the “scatterplot3d” package to visualize the spatial distribution of high-risk and low-risk groups, utilizing expression profiles of all genes, senescence-related genes, senescence-related lncRNAs, and risk-model-derived lncRNAs.

### 2.6. Evaluation of Tumor Microenvironment and Immune Cell Infiltration

Tumor microenvironment analysis was performed using the R packages “limma”, “estimate (implementing the ESTIMATE algorithm)”, and “ggpubr”. Single-sample gene set enrichment analysis (ssGSEA) via the GSVA package was employed to evaluate differences in immune cell infiltration and immune-related functional activities between the high-risk and low-risk groups within the entire cohort. The results were visualized using box plots generated using the “ggpubr” package.

### 2.7. Drug Sensitivity Analysis

Drug sensitivity analysis can screen out the most effective drugs for patients through in vitro experiments or computational models, thereby avoiding the use of ineffective or highly toxic drugs. In this study, we conducted a comprehensive drug sensitivity analysis across the entire dataset using the R packages limma, ggpubr, and oncoPredict, with a significance threshold of *p* < 0.001, to predict the differential sensitivity of candidate drugs between the two risk subgroups of colon cancer patients.

### 2.8. Statistical Processing

Utilizing R language (version 4.2.2), we conducted LASSO Cox regression, survival analysis, and PCA. Kaplan–Meier analysis was performed with the “survival” package. Furthermore, the prediction model was validated using the “survival”, “pheatmap”, and “ggpubr” packages. Patients were stratified into distinct groups based on their risk scores and consistency analysis. For normally distributed data, *t*-tests were employed to assess intergroup differences, while non-parametric instability tests (e.g., the Mann–Whitney U test) were applied to non-normally distributed datasets. The chi-square test was utilized to compare categorical variables between the training and validation cohorts. Statistical significance was defined as a two-tailed *p*-value < 0.05 unless otherwise specified.

## 3. Results

### 3.1. Construction of a Prognostic Model for Senescence-Related lncRNA Results

**Figure 1:**
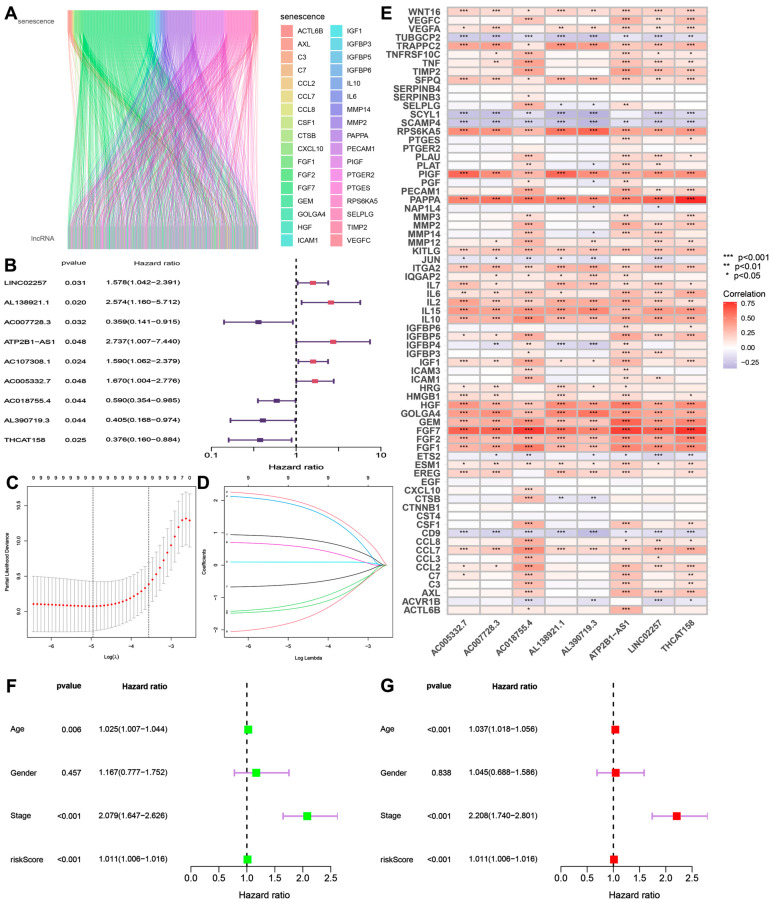
Construction of a prognostic model for senescence-associated lncRNAs. (**A**). Senescence-associated lncRNA Sankey diagram; (**B**) identification of senescence-associated lncRNAs with prognostic relevance through Pearson correlation analysis and survival modeling; (**C**,**D**) LASSO regression analysis and multifactorial Cox regression analysis; (**E**) correlation heatmap of senescence-associated genes; (**F**) one-factor Cox regression analysis; (**G**) multifactorial Cox regression analysis.

We randomly divided colon cancer patients into a training set (n = 182) and a testing set (n = 273). Table 1 shows the clinical and pathological characteristics of the patients. There was no statistically significant difference in the clinical characteristics between the training and testing sets (*p* > 0.05). Pearson correlation analysis identified 986 senescence-related lncRNAs related to aging genes, as shown in Figure 1A. The single-factor Cox regression analysis of the training set showed a total of nine lncRNAs strongly associated with disulfide death, and the forest plot of their risk values is shown in Figure 1B. LASSO regression analysis and multivariate Cox regression analysis further screened out eight lncRNAs associated with aging (Figure 1C,D). The correlation heatmap of senescence-related genes showed that senescence-related lncRNAs were positively correlated with the expression of 18 genes, including WNT16, IL-2, and FGF2, and negatively correlated with TUBGCP2, SCAMP4, and CD9, as shown in Figure 1E.

### 3.2. Independent Analysis of Prognostic Factors

**Figure 2:**
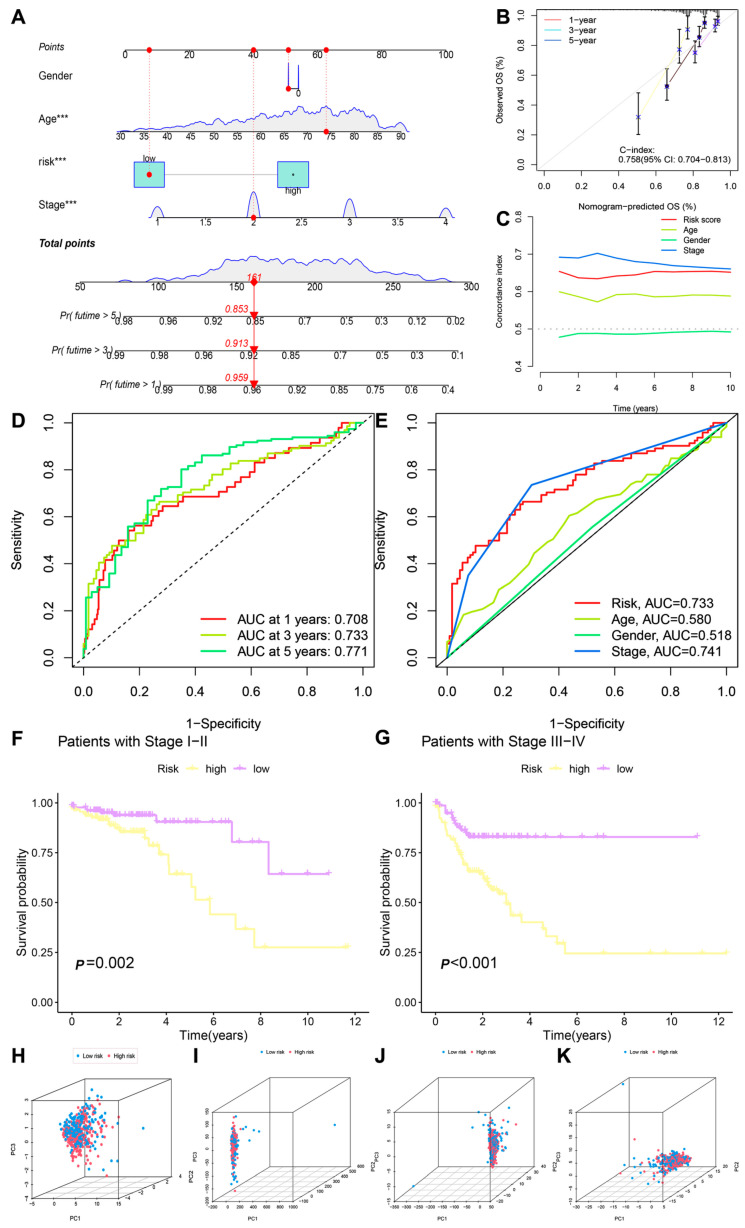
Validation of predictive models and survival analysis. (**A**,**B**) Column plots, ***: *p* < 0.001; (**C**) C-index; (**D**,**E**) ROC curves; (**F**,**G**) early- and late-survival KM analyses; (**H**) risk model lncRNA principal component analysis; (**I**) senescence-associated lncRNA principal component analysis; (**J**) senescence-associated gene principal component analysis; (**K**) principal component analysis of all genes.

To verify whether the senescence-related lncRNA prognostic model serves as a prognostic factor independent from other clinical traits, we performed an independent prognostic analysis on eight senescence-related lncRNAs along with other clinical features used in the model. The results of univariate and multivariate Cox regression analysis showed that risk score is an independent prognostic factor compared to other clinical features (Figure 1F,G). By combining risk scores with clinical pathological features, a column chart was used to predict 1-, 3-, and 5-year survival rates, with the predictive factors used including risk score, age, tumor grade, and staging. A calibration chart shows that the constructed model is close to the ideal model (Figure 2A). The C-index curve shows that the C-index of this feature is much higher than that of age and gender, indicating that the model has the highest accuracy in predicting patient survival (Figure 2B,C). In addition, the ROC curve shows that the model has high accuracy in predicting patient survival rates at 1 year, 3 years, and 5 years (AUC of 0.708, 0.733, 0.771) (Figure 2D), and its predictive ability is relatively high compared to other clinical features (AUC = 0.733) (Figure 2E). The above results indicate that the model can serve as an independent predictor and that it has stronger predictive ability than other clinical features. In order to test the predictive ability of the model for the prognosis of patients with different clinical features, survival analysis was conducted on patients with early- and late-stage tumors according to the risk group (high or low). The results show that the OS rate of the high-risk group was shorter than that of the low-risk group in both patients with early- and late-stage cancer (Figure 2F,G). PCA was performed using the expression levels of all genes, including senescence-related genes, senescence-related lncRNAs, and risk model lncRNAs. The results show that risk model lncRNAs more effectively distinguished between the high-risk and low-risk groups (Figure 2H–K).

### 3.3. Survival Analysis of Prognostic Models

**Figure 3:**
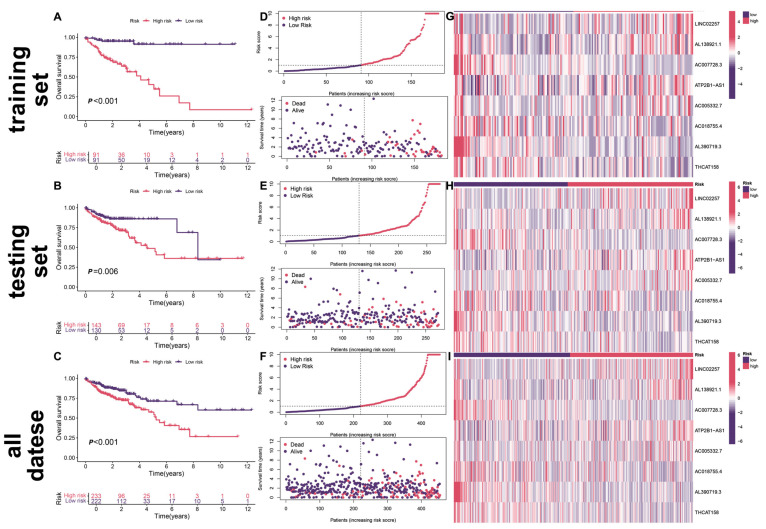
Survival status and risk scores for the total dataset, test set, and training set: (**A**–**C**) Kaplan–Meier survival analyses; (**D**–**F**) scatterplots of the distribution of RiskScore values and survival status for colon cancer samples; (**G**–**I**) heatmaps of differential expression of aging-associated lncRNAs.

In order to investigate the prognostic evaluation ability of the model, the same method as that used for the training set was used to calculate the risk score for each patient in the test set. Then, based on the median risk score of the training set, all patients in the dataset, training set, and testing set were divided into high-risk and low-risk groups. Survival analysis showed that in the three datasets, patients in the high-risk group had higher mortality rates and shorter OS compared to those in the low-risk group (Figure 3A–C), indicating that the senescence-related lncRNA prognostic model has strong predictive ability for colon cancer prognosis. The risk curve shows that the mortality rate for high-risk patients is higher than that for low-risk patients (Figure 3D–F). The heatmap shows the differential expression of senescence-related lncRNAs in the two groups, with LINC02257, AL138921.1, ATP2B1-AS1, and AC005332.7 highly expressed in the high-risk group and AC007728.3, AC018755.4, AL390719.3, and THCAT158 highly expressed in the low-risk group (Figure 3G–I).

### 3.4. Functional Enrichment Analysis

**Figure 4:**
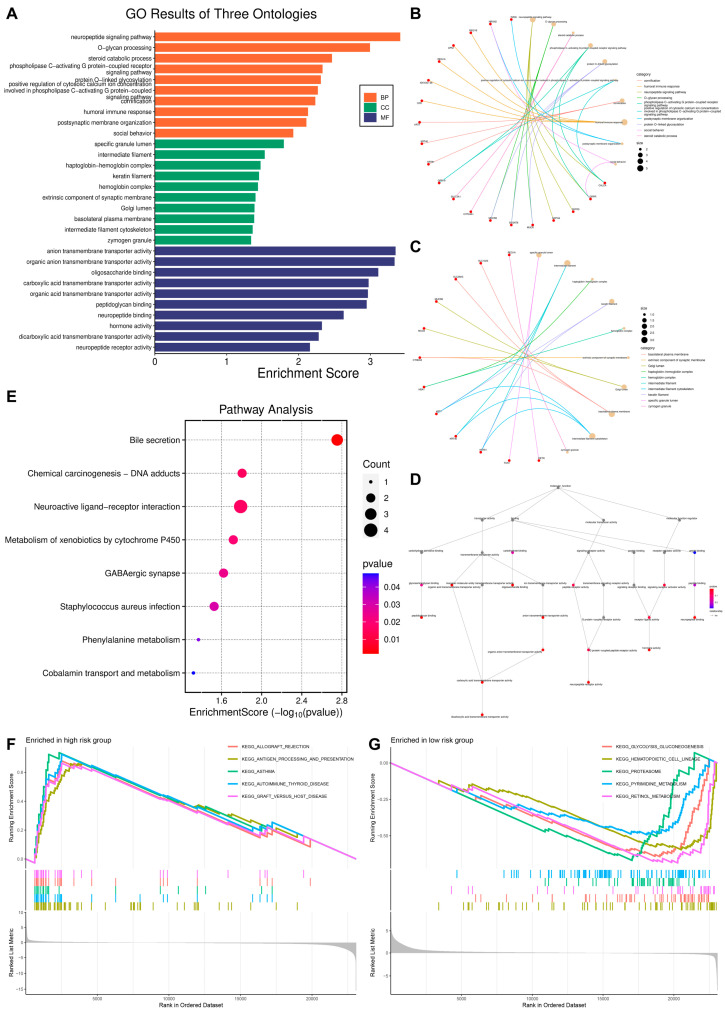
Enrichment analysis: (**A**) GO enrichment analysis bar graph; (**B**) network diagram showing specific enrichment in biological processes; (**C**) network diagram showing specific enrichment in cellular components; (**D**) network diagram showing specific enrichment in molecular functions; (**E**) KEGG pathway enrichment analysis; (**F**) GSEA enrichment in the high-risk group; (**G**) GSEA enrichment in the low-risk group.

Gene Ontology (GO) is an internationally standardized classification system of gene functions (Figure 4A). The GO biological process terms were mainly enriched in the neuropeptide signaling pathway, O-glycan processing, and steroid catabolic process (Figure 4B). The GO cellular component terms were mainly enriched in the specific granule lumen, intermediate filament, and haptoglobin–hemoglobin complex (Figure 4C). The GO molecular function terms were mainly enriched in anion transmembrane transporter activity, organic anion transmembrane transporter activity, and oligosaccharide binding (Figure 4D). Meanwhile, the Kyoto Encyclopedia of Genes and Genomes’ (KEGG) biological process terms were mainly enriched in bile secretion, chemical carcinogenesis—DNA adducts, and neuroactive ligand–receptor interaction (Figure 4E). GSEA and KEGG pathway enrichment analysis based on the high-risk group showed that the main pathways were allograft rejection and antigen processing and presentation (Figure 4F), while the low-risk group biological process terms were mainly enriched in glycolytic glycogenesis and pyrimidine metabolism (Figure 4G).

### 3.5. Immune Microenvironment Analysis and Drug Sensitivity Analysis

**Figure 5:**
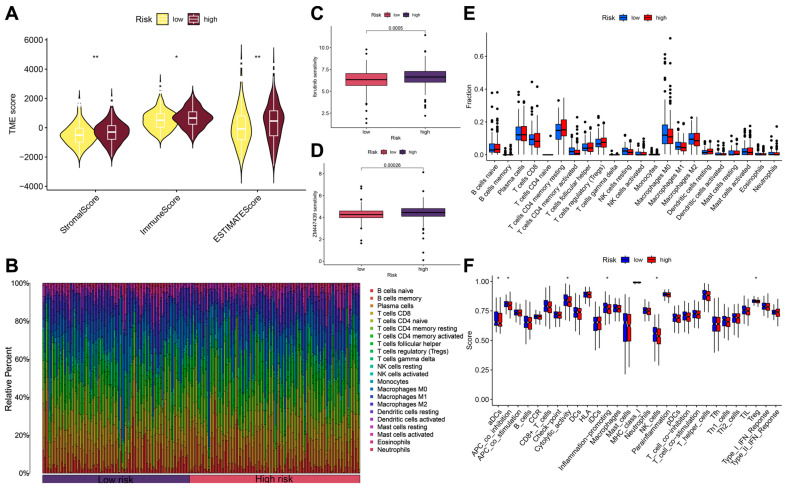
Immune infiltration and drug sensitivity analysis: (**A**) differences in tumor microenvironment between the high- and low-risk groups; (**B**) proportion of immune cells in high- and low-risk patients; (**C**,**D**) drug sensitivity analysis between patients in the high- and low-risk groups; (**E**) results of immune cell infiltration analysis; (**F**) results of the analysis of differences in immune function; *: *p* < 0.05; **: *p* < 0.01.

The results of the tumor microenvironment analysis showed that the immune score, stromal score, and total score of high-risk OS patients were higher than those of low-risk patients (Figure 5A). Memory B cells, M2-type macrophages, and activated dendritic cells were present at higher proportions in patients in the high-risk group (Figure 5B). Patients in the high-risk group exhibited higher sensitivity to selective ATP-competitive Aurora A inhibitors and ibrutinib (Figure 5C,D), indicating the therapeutic potential of these drugs. However, there was no significant difference in immune cell infiltration between the high- and low-risk patient groups (Figure 5E). Nevertheless, patients in the high-risk group had lower immune function, such as the co-inhibition of antigen-presenting cells, proinflammatory cells, and immune cell therapy (Figure 5F).

## 4. Discussion

Aging represents a cellular program characterized by the stable growth arrest of damaged or senescent cells. During the growth and development of organisms, processes such as normal embryonic development, tissue remodeling, and wound healing are essential. Tumor cells are also capable of undergoing aging. A key feature of tumor cells is their replication immortality and unchecked proliferation, which suggests that these cells have circumvented the aging processes induced by oncogenes and telomere shortening [10]. A recent study highlighted the potential of using anti-aging therapies (known as TIS) to treat colon cancer; lower doses of these agents are required compared to drugs that induce cancer cell apoptosis [11].

Previous models for predicting the influence of senescence-related genes in colon cancer have been established using bioinformatics methods [12]. This study established a scoring formula and chemotherapy drug sensitivity model for predicting the prognosis of colon cancer based on senescence-related lncRNA expression levels using bioinformatics methods. Combined with the lncRNA model, the efficacy of chemotherapy drugs was validated, providing a reference for precision medication and prognosis analysis strategies for colon cancer patients.

This study identified eight senescence-related lncRNAs and constructed a prognostic model through co-expression, LASSO regression, and Cox univariate and multivariate regression analyses. The results from the ROC curve, survival analysis, column chart, and heatmap showed that these eight senescence-related lncRNAs can accurately distinguish the prognosis of high-risk and low-risk patients. Moreover, the model using these lncRNAs has been validated using early- and late-stage patients and reliably predicts colon cancer patient outcomes. In addition, these lncRNAs are prognostic factors that are independent of other clinical features such as gender and age. Among the eight senescence-related lncRNAs in the prognostic model, LINC02257 was previously considered a key lncRNA in the prognosis of colon cancer and plays a critical role in the proliferation and metastasis of colon cancer cells through the LINC02257/JNK axis [13,14,15]. AL138921.1 plays an important role as a key factor in m6A methylation in N staging, survival time, and the immune landscape of colon cancer [16]. Previous studies have reported that ATP2B1-AS1 is an important lncRNA associated with disulfide-mediated cell death, playing a critical regulatory role in colon cancer, lung adenocarcinoma, and esophageal squamous cell carcinoma [17,18,19]. It is interesting that AC007728.3 and ATP2B1-AS1 are both lncRNAs that are involved in disulfide-mediated cell death in colon cancer [17]. Unfortunately, there are currently no reports involving research on THCAT158, AL390719.3, AC005332.7, and AC018755.4; investigating these lncRNAs will be a future research topic.

In summary, this study explored and identified senescence-related lncRNA biomarkers that can be used to predict the prognosis of colon cancer. However, there are still many shortcomings in this study. Firstly, the research data come from the TCGA database, and these retrospective data may have selection bias. Therefore, we also need to validate these results in large multi-center studies. In addition, in order to verify the pathological mechanism of colon cancer, in-depth research on predicting the efficacy of chemotherapy drugs based on the established model should be conducted through in vitro cell or model reliability testing. In summary, this study explored the potential interaction between lncRNA and aging to identify potential prognostic markers and search for predictive and therapeutic targets in colon cancer.

## Figures and Tables

**Table 1:** Comparison of clinical data between test and training sets.

Covariates	Typ	Total	Test	Train	*p*-Value
Age Age	≤65	189 (41.54%)	114 (41.76%)	75 (41.21%)	0.9845
	>65	266 (58.46%)	159 (58.24%)	107 (58.79%)	
Gender Gender	Female	216 (47.47%)	131 (47.99%)	85 (46.7%)	0.8631
	Male	239 (52.53%)	142 (52.01%)	97 (53.3%)	
Stage	Stage I	74 (16.26%)	47 (17.22%)	27 (14.84%)	0.8117
	Stage II	176 (38.68%)	106 (38.83%)	70 (38.46%)	
	Stage III	128 (28.13%)	73 (26.74%)	55 (30.22%)	
	Stage IV	65 (14.29%)	40 (14.65%)	25 (13.74%)	
	unknown	11 (2.42%)	6 (2.2%)	5 (2.75%)	
T	T1	11 (2.42%)	8 (2.93%)	3 (1.65%)	0.791
	T2	77 (16.92%)	44 (16.12%)	33 (18.13%)	
	T3	310 (68.13%)	187 (68.5%)	123 (67.58%)	
	T4	56 (12.31%)	33 (12.09%)	23 (12.64%)	
	unknown	1 (0.22%)	1 (0.37%)	0 (0%)	
M	M0	333 (73.19%)	199 (72.89%)	134 (73.63%)	0.8971
	M1	65 (14.29%)	40 (14.65%)	25 (13.74%)	
	unknown	57 (12.53%)	34 (12.45%)	23 (12.64%)	
N	N0	267 (58.68%)	165 (60.44%)	102 (56.04%)	0.3945
	N1	105 (23.08%)	57 (20.88%)	48 (26.37%)	
	N2	83 (18.24%)	51 (18.68%)	32 (17.58%)	

## Data Availability

All GWAS summary data that support the findings of this study are openly available in the TCGA and GEO databases.
